# Comparing western (*Megascops kennicottii*) and whiskered (*M. trichopsis*) screech-owl microbiomes in southern Arizona using a novel 16S rRNA sequencing method

**DOI:** 10.1186/s42523-022-00196-7

**Published:** 2022-07-30

**Authors:** Andrew W. Bartlow, S. Kane Moser, Jeremy E. Ellis, Charles D. Hathcock, Jeanne M. Fair

**Affiliations:** 1 Biosecurity and Public Health Group, Los Alamos National Laboratory, MS M888, Los Alamos, NM 87545 USA; 2 Fry Laboratories, LLC, Scottsdale, AZ 85260 USA; 3 Environmental Stewardship Group, Los Alamos National Laboratory, Los Alamos, NM 87545 USA

**Keywords:** Gut microbiota, Nestlings, Next generation sequencing, Microbial communities, Wildlife

## Abstract

Microbiomes are essential to a host’s physiology and health. Despite the overall importance of microbiomes to animal health, they remain understudied in wildlife. Microbiomes function as physical barriers to invading pathogens, and changes in the diversity or composition of microbes within a host may disrupt this barrier. In order to use microbiomes in wildlife ecology, knowledge of the natural variation within and among species is essential. We compare the diversity and composition of two avian species that share the same habitat and niche in our study area, the western screech-owl (*Megascops kennicottii*) and the whiskered screech-owl (*M. trichopsis*). We used a targeted 16S sequencing method to improve the taxonomic resolution of microbiomes. We found similar measures of alpha diversity between species and sample types (cloacal samples vs. fecal samples). However, there were significant differences in bacterial species richness among nestlings from different nest boxes, and the composition differed between the two bird species and among nestlings from different nest boxes. Western screech-owls had more variation in alpha diversity and composition and had fewer bacterial species in their core microbiome than whiskered screech-owls. Siblings are likely to yield similar findings for microbiomes; thus, sampling nestlings from different nests may be most informative for monitoring population-level changes.

## Introduction

Communities of microorganisms within animal hosts, collectively known as microbiomes, are known to play a key role in the behavior, health, and physiology of hosts [1]. The host organism constitutes a complex and variable environment, providing habitat types that suit a wide range of microbes, from the exposed surface of skin to the intestinal tract. The different microbial communities vary in the way they influence the overall health of their host. For example, the gut microbiome in particular has been shown to contribute significantly to host physiology and metabolism [1, 2]. Despite the known benefits and importance of microbiomes, one area of microbiome research that has had less attention historically is that of wildlife, especially in the context of evolutionary biology and conservation [3, 4]. Much remains unknown about the degree to which they influence their host’s ecology and evolution, and vice versa.

Characterizing microbiomes of wildlife is an essential step to better understanding the ecology, evolutionary history, and distributions of wildlife species [3, 4]. Wildlife microbiome diversity and composition vary naturally depending on environmental factors, which has implications for species conservation and disease ecology [4, 5]. Geographical location, fluctuations in climate, and niche differences, including habitat and food resources, all affect the microbiota of wild vertebrate species [6–9]. When the microbiome is greatly disrupted, resulting in dysbiosis, it can directly increase the risk of disease in animals and subsequently in humans [10–12]. Dysbiosis is most often caused by external factors, such as changes in host habitat, environmental change, and stress [5, 13]. Important signatures of dysbiosis are altered microbial composition and diversity (i.e., measures of alpha diversity and beta diversity) [10, 14, 15].

It has recently been proposed that the impacts of environmental change, including climate change, on wild host species may depend on the responses of their microbiomes [4]. For example, in coral reef ecosystems, microbiomes are essential for efficient nutrient cycling and preventing invasive species from colonizing [reviewed in 16. Changes in microbial composition due to rising seawater temperatures appear to precede visible signs of an impending bleaching event [6, 9]. In amphibians, which have notoriously sensitive skin microbiomes, shifts in microbial diversity can result in individuals being more susceptible to disease. Specifically, environmental change could alter defensive microbial species available in the environment to colonize amphibian skin [15]. A major benefit of microbial communities on, and in, hosts is their ability to limit colonization and establishment of pathogens [1, 17, 18].

A host’s microbiome functions as a physical barrier to pathogens trying to infect the host [1]. These microbial species can outcompete incoming pathogens and some produce antimicrobial compounds, resulting in the inability of pathogens to establish within the host [1, 19]. A decrease in the number of microbial species and changes to the composition of species within a host means that the host loses the benefits of the protective barrier and are potentially more vulnerable to pathogens [15]. Reliable information about microbe-host interactions, such as those microbes that are endemic to hosts and those that are not, could aid in earlier detection of threats, including the assessment of disease risk.

Recent advances in high-throughput sequencing have facilitated an explosion of research on microbiomes; however, there is still little data available on those of wildlife. If we hope to use wildlife microbiomes to inform conservation and disease mitigation efforts, the natural variation in microbial communities needs to be understood. This requires detailed and affordable surveillance methods as well as analyses that are relatively fast. The 16S rRNA gene is the most common target for classification of complex host and environmental samples. 16S metagenomic methods tend to target one region of the gene and bins reads into operational taxonomic units (OTUs). There are several drawbacks to using conventional 16S methods, including correcting for many 16S gene copy numbers, clustering OTUs, and differentiating among closely related species [20–23]. Here, we characterize and compare the microbiomes of two avian species by using a 16S method that uses an amplification strategy targeting two separate loci (variable regions 1 to 2 and 4 to 5) aiming to improve taxonomic identification resolution and copy number correction that is well suited for fecal microbiome analysis [24, 25]. By using two loci, this method can distinguish among closely related bacterial species. This method does not implement OTU-based identification, which can remove species-specific single nucleotide differences depending on the threshold selected for OTU generation. All unique reads are compared to the current NCBI NT and 16S Microbial databases, rather than OTU consensus sequences.

We examine the cloacal and fecal microbiomes of two avian species with overlapping habitat and food resource niches, the western screech-owl (*Megascops kennicottii*) and the whiskered screech-owl (*M. trichopsis*). The microbiomes of both species have never been described, and the whiskered screech-owl has not been as widely studied as the western screech-owl. This work will help in understanding the natural variation in wildlife microbiomes and how they differ in congeneric wild animal species with similar habitat niches [3]. Our study will also improve the paucity of data on wildlife microbiomes and inform sampling design strategies in the future for using microbiome data for conservation and threat assessment purposes. We had two main objectives. First, we sought to understand the similarities and differences in the microbiome of nestlings of each species given their shared range and shared habitat in our study area. Specifically, we aimed to compare alpha diversity and bacterial species composition between the two bird species and between two different sample types (cloacal and fecal samples). We also wanted to identify species that make up the core microbiome. Our second goal was to assess the utility of a new 16S rRNA next generation sequencing (NGS) method that aims to provide improved taxonomic resolution. This is the first application of this method to wildlife microbiomes.

## Methods

### Study location

**Fig. 1:**
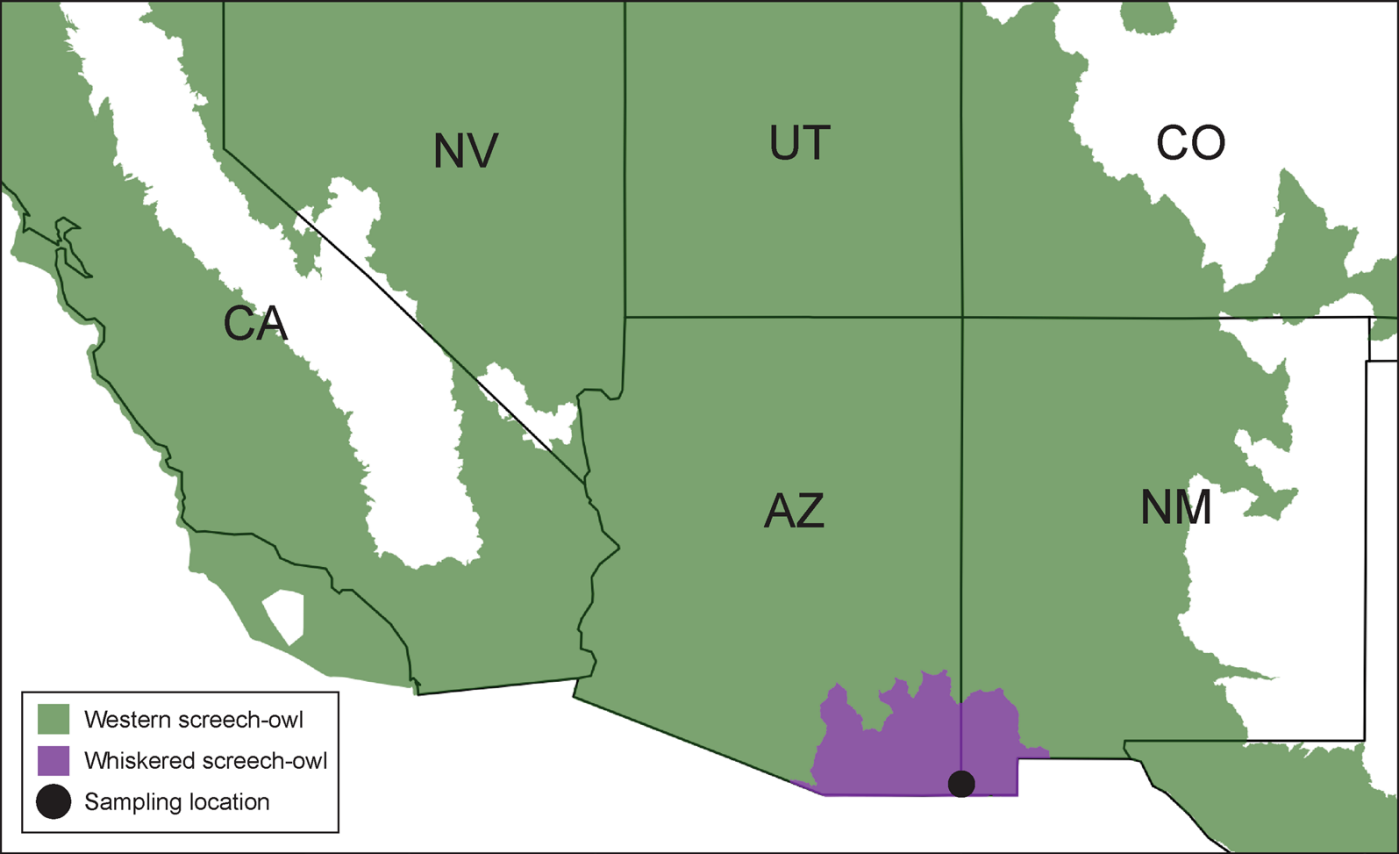
Distributions of the western screech-owl (*Megascops kennicottii*; green) and whiskered screech-owl (*M. trichopsis*; purple) in the United States. The sampling location in the Peloncillo Mountains in southeastern Arizona is marked with a black circle

This study was completed during May and June of 2019 and 2020 as part of a larger long-term study that has been running since 2015. Field work for this study was conducted in the Peloncillo Mountains in southeastern Arizona, near the borders of New Mexico and Mexico (31.4818°N, -109.0545°W, WGS 84). The distributions of western and whiskered screech-owls overlap in this region within the United States (Fig. 1). The distribution of the western screech-owl is found throughout much of the western United States and extends down into Mexico. The whiskered screech-owl is mainly found in Mexico and Central America, with the northern part of its range extending into southeastern Arizona and southwestern New Mexico.

### Sampling

Wooden screech-owl sized nest boxes were placed on trees to gather information on the ecology and life history traits of the two species. The interior dimensions of the nest boxes were 24.1 cm × 28.5 cm × 35.6 cm (length × width × height) and the entrance hole was 7.6 cm in diameter. All 15 nest boxes in this study were in the same canyon and in the same habitat. Nest boxes were checked during the early part of the breeding season (April–May) for the presence of eggs. Active nests were monitored until fledging. Nestlings were hand captured in the nest box when they were old enough (~ 20 days old) to be banded and to collect a blood sample for sexing. Blood samples were sent to an outside laboratory (Animal Genetics Inc. [USA]; https://www.animalgenetics.us/Avian/Avian-Index.asp) for DNA sexing for nestlings and adults. Adults were either hand captured at the nest box or captured using mist-nets around the nest box at night. All owls were banded with United States Federal Bird Banding Laboratory aluminum bands and morphological measurements recorded for adults. Some adults were alternatively sexed using breeding characteristics or morphology. Each nestling and adult sampled was placed in a clean mesh bird bag; we did not reuse bags. In total, we collected 29 samples from 16 individual birds (Table 1). Cloacal swabs were collected from western screech-owls (n = 9) for both nestlings (n = 7) and adults (n = 2) during this time using sterile flocked swabs (PurFlock Ultra, REF 25-3316-U). For whiskered screech-owls, we collected cloacal swabs (n = 7) from both nestlings (n = 4) and adults (n = 3). Fecal samples were collected by taking the entire fecal sacs. We only collected fresh feces from owls that defecated during handling, and they only came in contact with the clean bird bag material. All fecal samples were collected within 5 min of the owl defecating. Fecal samples were collected from western screech-owls (n = 6) from both nestlings (n = 5) and adults (n = 1). For whiskered screech-owls, we collected fecal swabs (n = 7) from both nestlings (n = 4) and adults (n = 3). For western screech-owls, nestlings were from 2 nest boxes, while whiskered screech-owl nestlings were from one nest box. Samples were immediately placed in a cooler with dry ice. The samples were transferred to a −80°C freezer within 2 days after initial collection. We collected fecal samples and swabbed the cloaca of all nestlings within a nest box.

**Table 1:** Metadata for each cloacal and fecal sample sequenced

Year sampled	Sample ID	Bird ID	Species	Sample type	Age	Sex	Box #	# of reads sequenced	Species with most reads (# reads)
2019	1954_001	1613–19835	Whiskered	Cloacal	Nestling	F	13	162,133	*Blautia hydrogenotrophica* (24,746)
2019	1954_036	1613–19835	Whiskered	Fecal	Nestling	F	13	88,290	*Faecalimonas umbilicata* (10,193)
2019	1954_003	1613–19834	Whiskered	Cloacal	Nestling	F	13	84,227	*Blautia hydrogenotrophica* (23,778)
2019	1954_004	1613–19834	Whiskered	Fecal	Nestling	F	13	110,217	*Blautia hydrogenotrophica* (24,023)
2019	1954_005	1613–19833	Whiskered	Cloacal	Nestling	F	13	108,648	*Blautia hydrogenotrophica* (22,192)
2019	1954_006	1613–19833	Whiskered	Fecal	Nestling	F	13	147,266	*Blautia hydrogenotrophica* (19,295)
2019	1954_007	1613–19832	Whiskered	Cloacal	Nestling	F	13	199,876	*Blautia hydrogenotrophica* (34,544)
2019	1954_008	1613–19832	Whiskered	Fecal	Nestling	F	13	139,362	*Faecalimonas umbilicata* (20,585)
2019	1954_009	844–74026	Western	Cloacal	Nestling	M	2	126,281	*Bifidobacterium cuniculi* (19,539)
2019	1954_010	844–74026	Western	Fecal	Nestling	M	2	25,896	*Bifidobacterium gallicum* (10,319)
2019	1954_011	844–74027	Western	Cloacal	Nestling	F	2	58,921	*Collinsella intestinalis* (8676)
2019	1954_012	844–74027	Western	Fecal	Nestling	F	2	207,890	*Drancourtella massiliensis* (16,196)
2019	1954_047	844–21695	Western	Cloacal	Nestling	U	2	15,651	*Absiella dolichum* (2120)
2019	1954_014	844–21695	Western	Fecal	Nestling	U	2	114,472	*Anaeromassilibacillus senegalensis* (18,815)
2019	1954_015	874–00013	Whiskered	Cloacal	Adult	F	13	80,968	*Collinsella intestinalis* (8314)
2019	1954_016	874–00013	Whiskered	Fecal	Adult	F	13	33,431	*Collinsella intestinalis* (4596)
2019	1954_017	1084–18726	Western	Cloacal	Adult	F	2	200,944	*Diplorickettsia massiliensis* (79,880)
2019	1954_018	1084–18726	Western	Fecal	Adult	F	2	83,713	*Diplorickettsia massiliensis* (32,425)
2020	1954_019	874–00014	Whiskered	Fecal	Adult	F	1	98,689	*Collinsella intestinalis* (21,750)
2020	1954_054	874–00014	Whiskered	Cloacal	Adult	F	1	3399	*Collinsella intestinalis* (313)
2020	1954_021	1613–19836	Whiskered	Fecal	Adult	F	6	9024	*Enorma timonensis* (737)
2020	1954_022	1613–19836	Whiskered	Cloacal	Adult	F	6	92,626	*Collinsella intestinalis* (21,044)
2020	1954_023	1084–18727	Western	Fecal	Nestling	F	12	91,118	*Blautia hydrogenotrophica* (17,551)
2020	1954_024	1084–18727	Western	Cloacal	Nestling	F	12	48,126	*Blautia hydrogenotrophica* (8189)
2020	1954_025	1084–18729	Western	Fecal	Nestling	M	12	197,382	*Bacteroides fluxus* (115,116)
2020	1954_026	1084–18279	Western	Cloacal	Nestling	M	12	155,414	*Blautia hydrogenotrophica* (23,423)
2020	1954_027	1084–18728	Western	Cloacal	Nestling	F	12	168,507	*Drancourtella massiliensis* (34,997)
2020	1954_028	1084–18730	Western	Cloacal	Nestling	F	12	62,440	*Corynebacterium falsenii* (8412)
2020	1954_029	874–00015	Western	Cloacal	Adult	M	12	77,508	*Lactobacillus pantheris* (20,992)

Nestlings from the same box are siblings, while adults from the same box are parents. Also listed is the number of reads sequenced after filtering for each fecal and cloacal sample and the bacterial species with the most reads identified

### DNA extraction and NGS sequencing

For each sample, approximately 200 µl nuclease free water was added to the sample and then DNA was extracted using the Zymo Quick Fungal/Bacterial Mini Prep Kit (Zymo Research, Cat. #D6005) and eluted in 50 µl elution buffer. The concentration of the DNA was obtained using the Qubit dsDNA HS Assay (ThermoFisher Scientific, Cat. #Q32854).

We performed amplicon-based sequencing utilizing a 16S sequencing approach that targets two independent taxonomically informative loci each containing two variable regions (16S variable regions 1 through 2 and regions 4 through 5). The ready-to-sequence amplicons for these two loci that contain sample index and adapters were generated using 2 µl of extracted DNA as template for the 16S Microbial ID Kit (BioID Genomics, Inc., Cat. #1000000). The concentration of the resulting amplicon pools was obtained using the Qubit dsDNA HS Assay (ThermoFisher Scientific, Cat. #Q32854). Additionally, the average size of the library was determined by the Agilent High Sensitivity DNA Kit (Agilent, Cat. #5067-4626). An accurate library quantification was determined using the Library Quantification Kit—Illumina/Universal Kit (KAPA Biosystems, Cat. #KK4824). No PhiX or library preparation procedures were required, and the diluted amplicon pool was sequenced on the Illumina MiSeq using the MiSeq Reagent Kit v3 (600-cycle) (Illumina, Cat. #MS-102-3003) to generate paired end 251 bp reads. This produced a single FASTQ file. Each sample was sequenced in duplicate to detect problems with amplification among samples. The 16S Microbial ID Kit comes with two amplification plates, each able to prepare and analyze 48 samples. We ran two no template control samples along with the owl samples, one for each plate.

### Bioinformatics

The subsequent FASTQ file was demultiplexed, paired-end merged, trimmed, and analyzed by the Rapid Infectious Disease Identification (RIDI) system (Fry Laboratories, LLC) [24, 26]. Low quality and mismatching base pairs in the overlapping paired-end regions are merged in the RIDI software, keeping the highest quality consensus. Flanking primer sequences are trimmed. Both of the 5’ and 3’ ends are trimmed, single bases at a time, until the end 5 base pairs exhibit Q30 or better. These resulting reads were identified to species level, or lowest applicable taxonomic level. The RIDI system compares all recovered putative prokaryotic sequences to both the NT and the 16SMicrobial NCBI databases. Microbial identification was reported as the nearest identified species for each sequence. Raw reads were tabulated per bacterial species and relative normalized cell abundances were calculated based on the copy number of the 16S rRNA gene for each species or identified taxon and the raw reads.

### Data analysis

All of the data processing and statistical analyses were performed using the statistical software program R (version 4.1.1 [27]). First, reads that were not able to be identified to species level were removed. This primarily included reads that could only be identified to genus level. Since each read is analyzed using a BLAST-based approached, it would not be appropriate to include reads that can only be identified to genus in our species-level analyses.

The results given were compiled into a phyloseq object using the phyloseq package (version 1.38.0 [28]) in R. Our phyloseq object included our species by sample matrix (rows as species) with read number in each cell, the sample metadata, and a taxonomy table in which the taxon ranks were listed for each bacterial species. For the majority of the analyses discussed below, we focused on cloacal (n = 11; n = 7 for western and n = 4 for whiskered) and fecal (n = 9; n = 5 for western and n = 4 for whiskered) samples from nestlings only due to sample sizes. We were not able to obtain fecal samples from two of the western screech-owl nestlings. Also, we did not compare males and females, because we only had two male nestlings (both western screech-owls). Because of unequal numbers of reads sequenced in our nestling samples, we rarefied all nestling samples to the lowest number of reads obtained for a single sample, which was 15,651. This rarefied dataset was used to compare two measures of alpha diversity.

Using the phyloseq package, we compared the abundance of bacterial phyla present in all 29 samples. We then compared two measures of alpha diversity (species richness and Shannon diversity) between the two owl species for each of the two sample types (cloacal samples and fecal samples) for nestlings only. To determine significant differences for each alpha diversity comparison, we used two-sample t-tests. The residuals of each t-test were normally distributed. To test for differences in variation in the observed richness and Shannon diversity between the two bird species, we used the F test. We compared the cloacal samples and fecal samples separately.

In order to compare bacterial species composition among our samples, we used non-metric multidimensional scaling (NMDS) using Bray–Curtis distances using the non-rarefied dataset. We transformed the abundance data into proportions for the ordination. We first compared species composition among the two owl species, the two sample types (fecal and cloacal), and nest boxes for nestlings only. We tested for differences in the dispersion (i.e., variances) between the various groups using the betadisper function in the vegan package. To determine significant differences for each variable, we used permutational ANOVAs (PERMANOVAs) using the adonis2 function in the vegan package (version 2.5.7 [29]) with 1,000 permutations. We tested bird species, sample type, and box in the same model. We included an interaction term between bird species and sample type. The interaction term was not significant, so results are reported from the model without the interaction term. We also ran NMDS with adults included to test whether adults clustered near their own nestlings and tested for differences between box type and age. Our full model included bird species, sample type, box, and age. For the adult samples, we only used cloacal samples and fecal samples from five adults from boxes 2, 12, and 13 (Table 1); adults from boxes 1 and 6 did not have any nestlings for comparisons.

The core microbiome was determined by using the microbiome package (version 1.16.0 [30]) in R. We used the core_members function to find the core bacterial species in both cloacal and fecal samples in each bird species separately and those bacterial species that are present in all types of samples for nestlings only. For western screech-owls (n = 12), we set the prevalence threshold to 50% and the detection level to 0.01, meaning that a given bacterial species had to be present in at least 6 of the samples and at a relative abundance of 0.01 or greater in each of those samples. Because there were less whiskered screech-owls (n = 8) than western screech-owls, we set the prevalence threshold of 75% and the detection level to 0.01. For whiskered screech-owls, a given bacterial species had to be present in at least 6 individuals. For all nestling samples, we used a prevalence threshold of 50% and the detection level to 0.01. We show the prevalence of the core bacterial taxa according to various detection threshold values.

## Results

We collected 16 cloacal samples and 13 fecal samples from 16 owls (n = 7 whiskered screech-owl, and n = 9 western screech-owl; Table 1). A total of 3,010,535 reads were produced from the 29 samples. These are the weighted reads based on the copy number of the 16S rRNA gene for each identified species or taxon. After filtering out genus level only identifications, we were left with 2,992,419 reads from 29 samples. Reads belonged to 3,404 total bacterial species. These results, and those in Table 1, include samples from adult owls (n = 9). The analyses that are based on only nestlings had 2,312,117 reads and a total of 2,819 total bacterial species, which means that in the 680,302 filtered out reads, there were 585 bacterial species only found in adults.

The first plate of the sequencing run generally produced a high number of reads for most samples. On the other hand, the second plate produced few reads for most samples due to reasons unknown. However, for three samples (1954_036, 1954_47, 1954_54), the first sequencing attempt produced few reads with poor quality results. Therefore, we used the results of the second sequencing run for these samples, which provided greater numbers of reads (Table 1). The results for each sample presented here are based on one sequencing run. In the two negative control samples, we found 56 total reads in one control and 30 reads in the second control. Almost all of these reads were singletons, and the identity of most of these contaminant species are the major contributing species in our samples. Thus, contamination likely plays a very minor role in the results shown here, especially given the high number of reads sequenced and the number of bacterial species identified in our samples.

### Taxonomic diversity

**Fig. 2:**
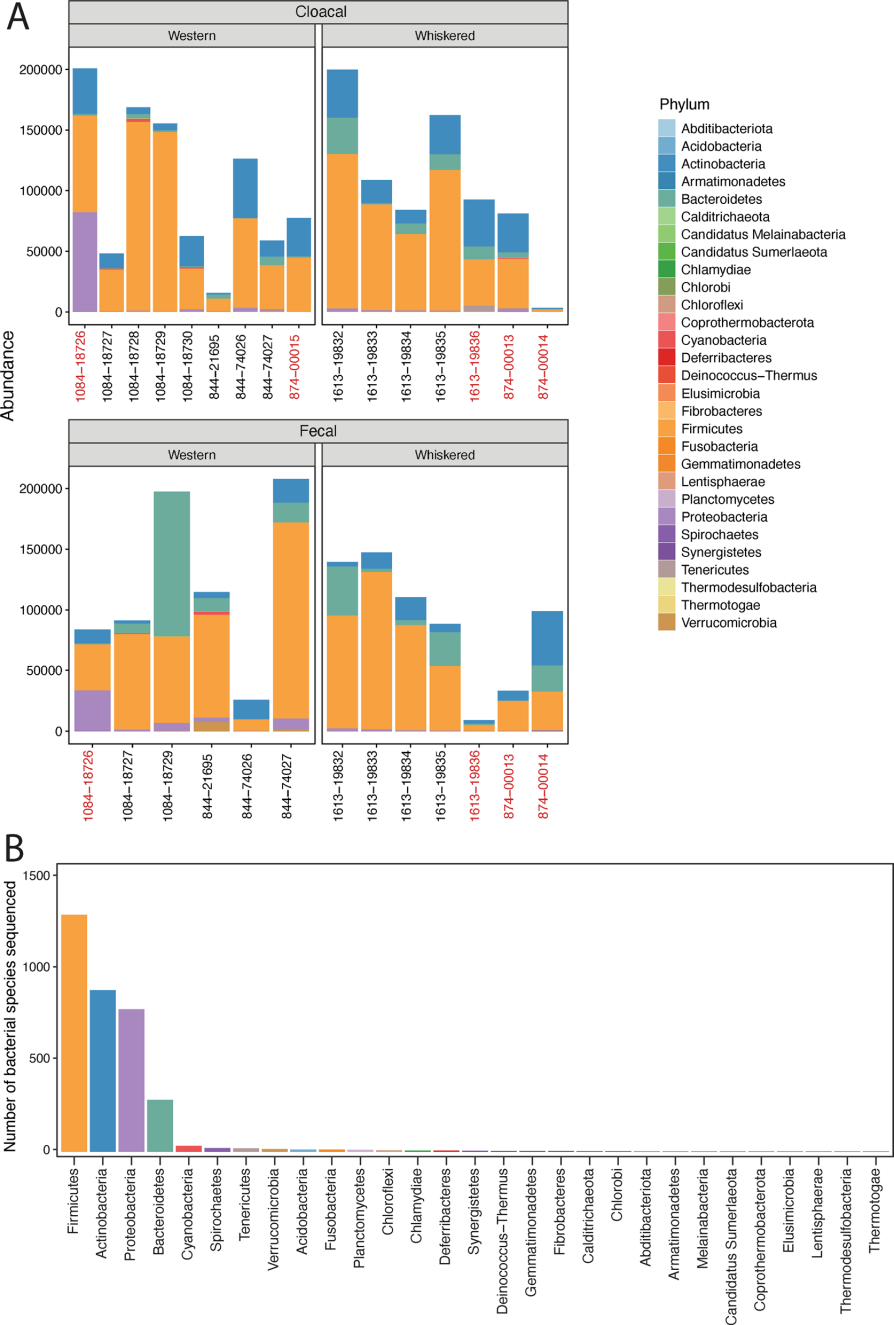
The number of reads (abundance) of each phylum present in samples from nestlings of western and whiskered screech-owls (**A**), and the total number of bacterial species in each of the phyla represented (**B**). Samples in (**A**) were split according to sample type (cloacal and fecal) and screech-owl species. Bird ID is on the X-axis and the red IDs denote adult birds

In all of our samples, bacteria species belonged to 29 phyla (Fig. 2A). Members of the following phyla were the most dominant and were consistently found in fecal and cloacal samples: Actinobacteria, Bacteroides, Firmicutes, and Proteobacteria (Fig. 2). We also found species belonging to the phyla Chlamydiae, Cyanobacteria, Fibrobacteres, Fusobacteria, Spirochaetes, Tenericutes, Verrucomicrobia, Acidobacteria, Chloroflexi, Thermodesulfobacteria, Chlorobi, Planctomycetes, Gemmatimonadetes, Deferribacteres, Deinococcus-Thermus, Candidatus Sumerlaeota, Melainabacteria, Coprothermobacterota, Elusimicrobia, Synergistetes, Thermotogae, Armatimonadetes, Abditibacteriota, Lentisphaerae, Calditrichaeota, although much fewer reads and species were identified from these phyla (Fig. 2B).

**Fig. 3:**
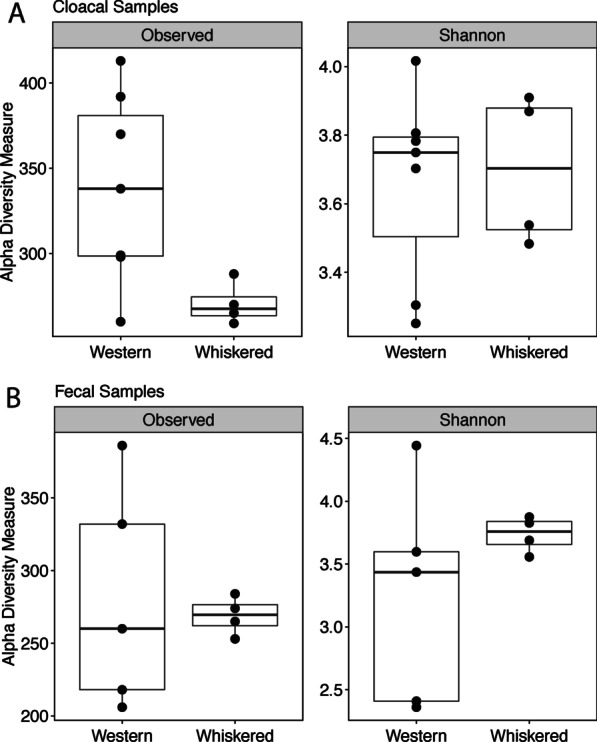
Observed richness and Shannon diversity for cloacal samples (**A**) and fecal samples (**B**) of nestlings of western and whiskered screech-owls. Black points denote the diversity measurements for each sample. Only bacterial species richness significantly differed between bird species for cloacal samples

In both types of samples, fewer reads were sequenced from whiskered screech-owls compared to western screech-owls. After rarefying our data to 15,651 sequences, we were left with 1357 bacterial species from our 20 nestling samples. Two measures of alpha diversity, species richness and Shannon diversity, were compared between bird species and sample type for nestlings only. Species richness significantly differed between the two bird species for cloacal samples (t-test: d.f. = 6.99, t = 3.09, *P* = 0.018; Fig. 3A) but not fecal samples (t-test: d.f. = 4.29, t = 0.33, *P* = 0.76; Fig. 3B). For cloacal samples, western screech-owls had a mean of 338.6 bacterial species, while whiskered screech-owls had a mean of 270.5 bacterial species. There was no significant difference in Shannon diversity between the two species for either the cloacal samples (t-test: d.f. = 7.75, t = -0.27, *P* = 0.80; Fig. 3A) or fecal samples (t-test: d.f. = 4.27, t = −1.22, *P* = 0.28; Fig. 3B). There were no significant differences in bacterial species richness between sample types for western screech-owls (t-test: d.f. = 6.93, t = 1.44, *P* = 0.19) and whiskered screech-owls (t-test: d.f. = 5.98, t = 0.17, *P* = 0.87), nor were there significant differences in Shannon diversity between sample types for western screech-owls (t-test: d.f. = 4.58, t = 1.01, *P* = 0.36) or whiskered screech-owls (t-test: d.f. = 5.16, t = −0.28, *P* = 0.79).

For fecal samples, there was more variation in the observed richness (F test: F = 33.99, *P* = 0.02) and Shannon diversity (F test: F = 37.34, *P* = 0.014) in western screech-owls compared to whiskered screech-owls. For cloacal samples, there was more variation in the observed richness (F test: F = 20.01, *P* = 0.03), but not Shannon diversity, in western screech-owls compared to whiskered screech-owls (F test: F = 1.6, *P* = 0.75).

There was a significant difference in bacterial species richness for nestlings in different nest boxes for both cloacal (ANOVA: F_2,8_ = 4.45, *P* = 0.05) and fecal samples (ANOVA: F_2,6_ = 5.55, *P* = 0.04). However, there was no significant difference in Shannon diversity for nestlings in different nest boxes for either cloacal (ANOVA: F_2,8_ = 2.45, *P* = 0.15) or fecal samples (ANOVA: F_2,6_ = 1.03, *P* = 0.41).

### Species composition

**Fig. 4:**
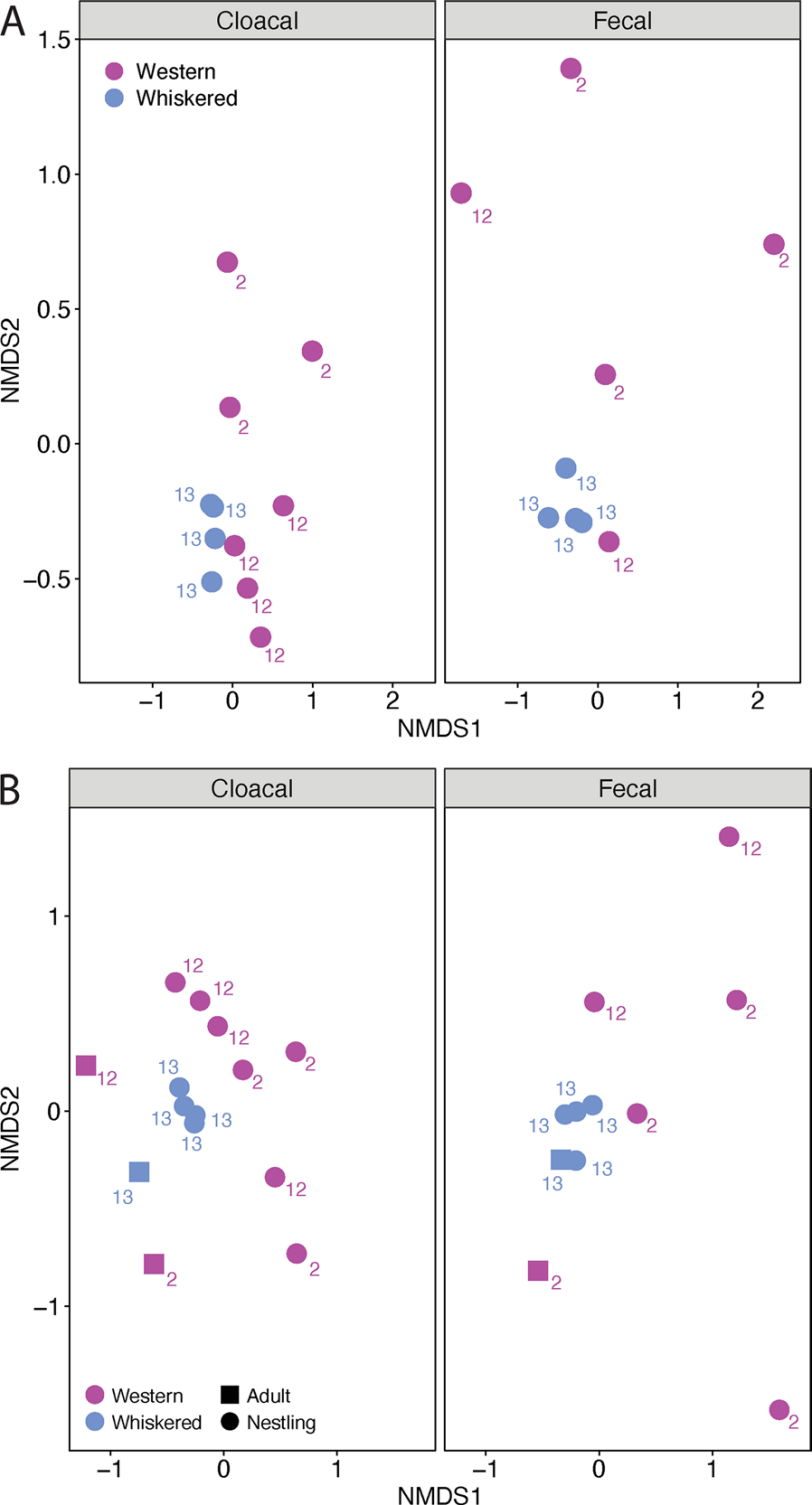
Non-metric multidimensional scaling (NMDS) of species composition using Bray–Curtis distances for nestlings of western and whiskered screech-owls (**A**) and with adults included (**B**). Within both A and B, cloacal and fecal samples are separated, and the scales for both panels are the same, making them directly comparable. Samples are labeled according to nest box number

We calculated Bray–Curtis dissimilarities and analyzed composition using NMDS ordination plots. For the NMDS with nestlings only, we used k = 2 and the final stress was 0.098. There was no significant interaction between bird species and sample type (R^2^ = 0.04, *P* = 0.43) in the initial full model. The full model with no interaction term with bird species, sample type, and nest box was significant and explained around 37.2% of the variation in bacterial species composition (R^2^ = 0.37, *P* < 0.001). The two bird species differed in composition from one another (R^2^ = 0.19, *P* < 0.001; Fig. 4A). Nestlings of the same species were more similar to their own nestmates (siblings) than to those in different nests (R^2^ = 0.12, *P* < 0.001; Fig. 4A). There was no significant difference between sample types (R^2^ = 0.06, *P* = 0.06), although this result is very close to being significant. Adding nestling ID to the model resulted in the model explaining 77.2% of the variation in microbiome composition (R^2^ = 0.77, *P* < 0.001).

We tested for differences in dispersion using the betadisper function. There were significant differences in dispersion between the two screech-owl species (ANOVA: F_1,18_ = 22.07, *P* < 0.001) and among nestlings from different nest boxes (ANOVA: F_1,18_ = 4.95, *P* = 0.02). Nest boxes 13 and 2 significantly differed in their dispersion (TukeyHSD: *P* = 0.02), while boxes 13 and 12 did not differ (TukeyHSD: *P* = 0.16), nor did boxes 12 and 2 (TukeyHSD: *P* = 0.56). There were no significant differences in dispersion between sample types (ANOVA: F_1,18_ = 0.82, *P* = 0.38).

For the NMDS analysis with adults added, we also used k = 2 and the final stress was 0.16. With adults added, we still found significant differences among nest boxes (R^2^ = 0.10, *P* < 0.001; Fig. 4B) and between bird species (R^2^ = 0.14, *P* < 0.001; Fig. 4B). Bacterial species composition also differed between adults and nestlings (R^2^ = 0.11, *P* < 0.001; Fig. 4B). There was no significant difference between sample types (R^2^ = 0.04, *P* = 0.10; Fig. 4B).

Similar to the nestling samples, there was a significant difference in dispersion for species (ANOVA: F_1,23_ = 26.9, *P* < 0.001) and among nestlings from different nest boxes (ANOVA: F_1,22_ = 5.95, *P* = 0.01). Nest boxes 13 and 2 significantly differed in their dispersion (TukeyHSD: *P* = 0.008). There was not a significant difference between boxes 13 and 12 (TukeyHSD: *P* = 0.10) or between boxes 12 and 2 (TukeyHSD: *P* = 0.57). There were no significant differences in dispersion between sample types (ANOVA: F_1,23_ = 0.23, *P* = 0.64) or age (ANOVA: F_1,23_ = 0.94, *P* = 0.34).

### Core microbiome

**Fig. 5:**
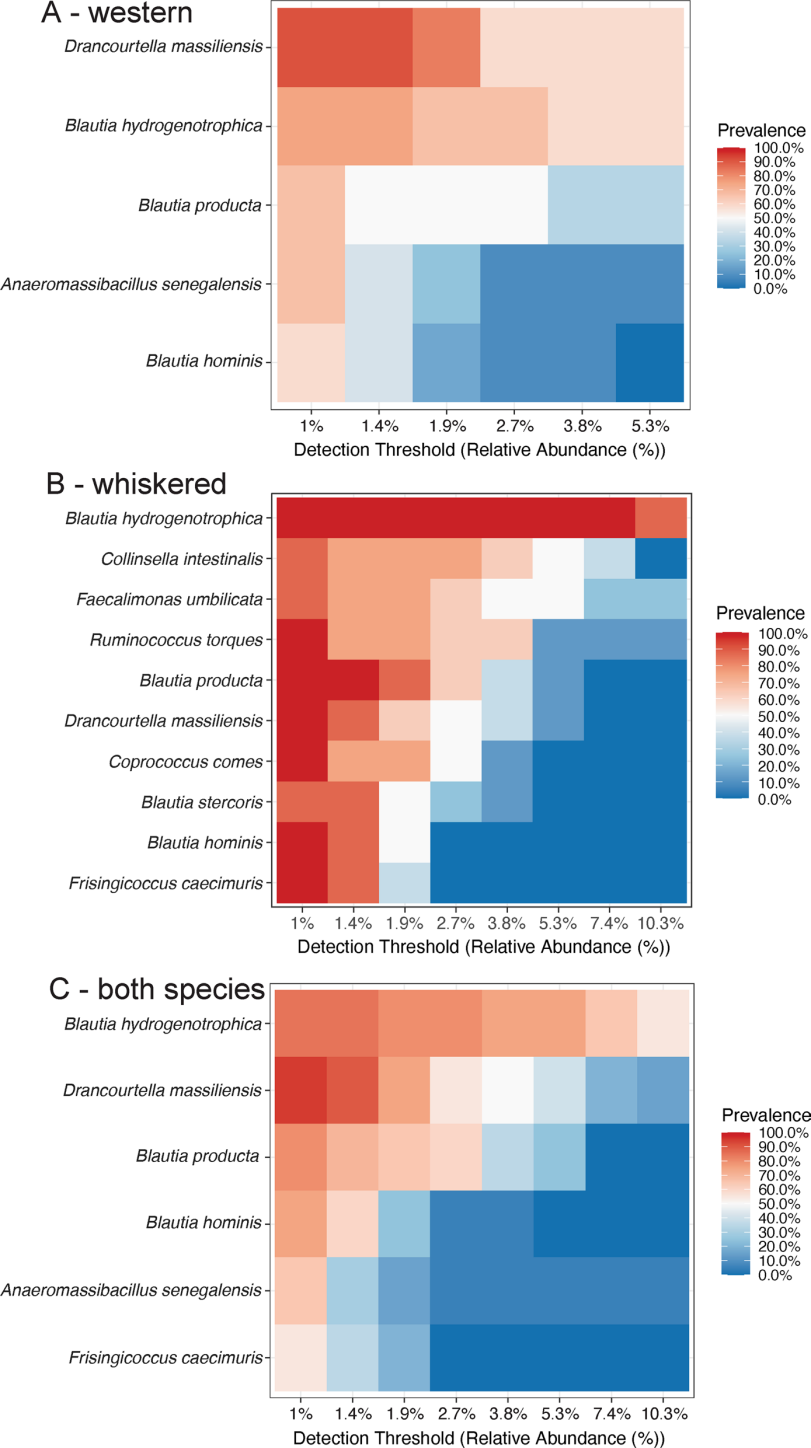
The core microbiome of both cloacal and fecal samples for western screech-owls (**A**), whiskered screech-owls (**B**), and both species combined (**C**). Shown are the detection thresholds for proportion of reads for varying prevalence levels. All bacterial species at the detection threshold of 1% have over 50% prevalence for western screech-owls and for both species. A 75% prevalence was used for whiskered screech-owls because of the smaller number of individuals sampled. Only a few bacterial species have greater than 50% prevalence at greater threshold percentages

We compiled the core microbiome for each bird species and for both species combined. The criteria used were that the bacterial species had to be found in at least 50% (western) or 75% (whiskered) of the samples (prevalence) at an abundance of 1% or greater in each of those samples. There were only five bacterial species that met the criteria for the western screech-owls (Fig. 5A). On the other hand, there were 10 bacterial species that met the criteria for whiskered screech-owls (Fig. 5B). For both bird species combined, there were six bacterial species that met our core microbiome criteria (Fig. 5C). Figure 5 also shows the prevalence of these bacterial species when different detection thresholds are used to define the core microbiome. There are only a few bacterial species that pass the 50% prevalence threshold at a detection threshold of 3% (Fig. 5). The species that is consistently the most abundant is *Blautia hydrogenotrophica.*

## Discussion

The interactions between host, microbiome, and environment are complex and likely play an important role in the evolutionary ecology of wildlife species. We examined the composition and alpha diversity of the fecal and cloacal microbiomes of two understudied avian species, western and whiskered screech-owls. These species have overlapping ranges and share the same habitat in our study area, and they will even nest in nest boxes that were previously used by individuals of the other species. Our goal was to provide insight into the natural variation in wildlife microbiomes and how they differ in congeneric species with similar habitat niches in the hope of adding to the scarcity of data on wildlife microbiomes and to inform sampling design strategies in the future.

We found relatively similar measures of alpha diversity between species and sample types. The only significant difference was that in cloacal samples bacterial species richness was significantly higher for western screech-owls compared to whiskered screech-owls. Nestlings from different nest boxes differed in species richness for both cloacal and fecal samples. Sample type was not significant, although a larger sample size may reveal differences in alpha diversity between cloacal and fecal samples. In terms of bacterial species composition, we found that bird species and nest box were correlated with microbiome composition. Samples grouped according to nest box, which may be due to similar types of food being fed to all the nestlings within a nest box. Additional work would be needed to test hypotheses related to diet as there is limited information about the diets of these owl species in this part of their respective ranges.

Small owls like screech-owls tend to eat mostly arthropods. The diet of western screech-owls in the Chiricahua Mountains, approximately 48 km north-northwest from our study area, is comprised of 82% arthropods [31–33]. Western screech-owl populations in southwestern deserts seem to take a higher proportion of invertebrates than northern populations do [34]. Being a smaller owl, the whiskered screech-owl diet is comprised of 85% to 90% arthropods [31, 32]. Regardless of their specific diets, our findings indicate that for characterizing both alpha diversity and species composition it is important to consider siblings within a nest or in a nest box when designing monitoring programs for use in conservation and threat assessments.

Several results suggest that there are consistent differences between the nestlings of these owl species regarding variation in their microbiomes. Using the rarefied dataset, there was significantly greater variation in cloacal and fecal sample bacterial species richness and fecal sample Shannon diversity in western screech-owls compared to whiskered screech-owls. Similarly, there is significantly greater dispersion in bacterial species composition in western screech-owls. Nest boxes also differed in their dispersion with box 13 (whiskered screech-owls) having the least amount of dispersion. It is not clear whether the significant differences in composition (using the adonis test) between owl species and among nest boxes are due to these differences in dispersion. However, all the samples from whiskered screech-owls grouped very close together in the plots of the NMDS ordinations compared to western screech-owls. Additionally, the adonis test seems to be the least sensitive to dispersion compared to similar tests [29]. These results could also be the result of different numbers of nestlings sampled in each nest box; all four of the whiskered screech-owl nestlings were from the same box. The western screech-owl nestlings were from two nest boxes, (i.e., box 12 had four nestlings and box 2 had three nestlings). However, even within each western screech-owl nest box, the samples do not cluster as close together as those from whiskered screech-owls.

Another result consistent with this pattern of variation differences is that the core microbiome in whiskered screech-owls has twice as many bacterial species that are shared among those nestlings than in western screech-owl nestlings. In order to control for sample size differences between owl species, we used a higher prevalence threshold for the whiskered screech-owl samples. Our sample sizes are small, so these results should be interpreted with caution.

When adults were added to the ordination analysis, these samples generally clustered together, rather than cluster near their nestlings. There were still significant differences in individuals from different nest boxes. These composition differences could be age-related differences in diet. The top species in both sample types in one adult bird (1954_18726) is *Diplorickettsia massiliensis* (Table 1). These samples are the only ones in which this species was the most abundant. This species was found in other samples as well, but in very low abundance; there were only 3 samples with more than 100 reads with the next highest containing 2,062 reads. This species was first isolated in ticks (*Ixodes ricinus*) in Slovakia [35] and is considered a tick-borne human pathogen found in blood [36]. A closely related species was recently found in the United States [37]. It is not known whether this adult bird was in poor condition when it was sampled or if it consumed ticks infected with the pathogen.

Very few microbiome studies have been done on owls (but see [38]). In a recent study, barn owls (*Tyto alba*) were shown to have sex differences in their microbiomes, with females having more diverse microbiomes than males [38]. It is unclear if there would be differences in these species if only adults were sampled. We could not test sex differences with our data with only 2 male nestlings being sampled. In the same study in barn owls, the authors found that owls that had larger foraging areas had more diverse microbiomes. Western screech-owls could have larger foraging areas, resulting in larger variation in their bacterial communities; however, no studies have assessed this in our study area. Future studies on sex/age differences and foraging ecology in these populations are warranted to understand how behavioral ecology structures microbiome-host interactions, and if similar patterns exist among different owl species.

*Blautia hydrogenotrophica* (formerly called *Ruminococcus hydrogenotrophicus* [39]) was consistently one of the most abundant species in many of our samples. This species, along with other species in the genus (specifically, *B. producta* and *B. hominis*), were also part of the core microbiomes identified. Most reports of *B. hydrogenotrophica* come from humans and ruminants, but several studies report this species in broiler chickens [40, 41], especially in context with potential probiotic properties [42]. This species is known to help the host digest plant material [42], which may point to these owls consuming herbivorous arthropods.

The 16S technology used here has been mainly used to detect bacterial pathogens in blood in human patients [24, 26, 43]. While targeted 16S sequencing has been used previously, this is the first use of this kit to characterize the microbiome of a wild animal species. We show that this method identified many bacterial species present in our samples and does not utilize binning similar sequences into OTUs before assigning species names. Our results suggest that this is a well suited method to characterize and compare microbiomes from both fecal and cloacal samples.

In general, either fecal or cloacal samples will be useful in characterizing the microbiomes of these species of screech-owls since there were few differences between sample types. However, this will depend on the research goals. For example, if the goal involves characterization of the colon microbiome, the fecal samples will likely be more appropriate [44]. Collecting fecal samples is less invasive than swabbing the cloaca, which is especially important to consider for small owl nestlings. Conversely, defecation within a bird bag will result in more haphazard sampling, given that some will not defecate in the time it takes to process them. It will also be harder to prevent sample contamination than swabbing. Here, we used clean mesh bird bags to prevent contamination and we also selected fresh fecal sacs within a few minutes of defecation. Regardless of the sample being used, siblings should be taken into the study design since both species richness and composition differ according to nest box.

A baseline understanding of the natural variability in wildlife microbiomes needs to be established for a given species or suite of species before we can begin to use these species as indicators of environmental change and potential threats from pathogens. Changes in the microbiome occur much faster than waiting for wildlife populations and communities to decline or show visible signs of an underlying threat. This could provide valuable information about host populations impacted by environmental change that would otherwise have to be obtained over much longer periods of time, which would make effective management strategies for mitigation and conservation less efficient and harder to implement. For these data to be useful for such a task, we need reliable baseline data on appropriate species and how microbiomes are structured within a species (e.g., sex, siblings, age, foraging range, etc.). For characterizing nestling microbiome studies, our results suggest that nest, either in a nest box or in a natural nesting cavity, be considered if nestlings are being sampled. Sampling nestlings from the same nest box would result in more similar microbiomes than sampling from multiple nest boxes. Sampling different nest boxes may be most informative for monitoring population-level changes in microbial communities. This factor could explain a portion of the variation in bacterial species composition in other avian species as well.

## Data Availability

All data will be uploaded to appropriate databases upon manuscript acceptance. Sequence data will be upload to the Sequence Read Archive (SRA).

## References

[1] Sekirov I, Russell SL, Antunes LCM, Finlay BB (2010). Gut microbiota in health and disease. Physiol Rev.

[2] Heijtz RD, Wang S, Anuar F, Qian Y, Björkholm B, Samuelsson A (2011). Normal gut microbiota modulates brain development and behavior. Proc Natl Acad Sci..

[3] Hird SM (2017). Evolutionary biology needs wild microbiomes. Front Microbiol.

[4] Trevelline BK, Fontaine SS, Hartup BK, Kohl KD (2019). Conservation biology needs a microbial renaissance: a call for the consideration of host-associated microbiota in wildlife management practices. Proc R Soc B Biol Sci.

[5] West AG, Waite DW, Deines P, Bourne DG, Digby A, McKenzie VJ (2019). The microbiome in threatened species conservation. Biol Conserv.

[6] Amato KR, Leigh SR, Kent A, Mackie RI, Yeoman CJ, Stumpf RM (2015). The gut microbiota appears to compensate for seasonal diet variation in the wild black howler monkey (Alouatta pigra). Microb Ecol.

[7] Gillingham MAF, Béchet A, Cézilly F, Wilhelm K, Rendón-Martos M, Borghesi F (2019). Offspring microbiomes differ across breeding sites in a panmictic species. Front Microbiol.

[8] Knutie SA, Chaves JA, Gotanda KM. Human activity can influence the gut microbiota of Darwin’s finches in the Galapagos Islands. Mol Ecol. 2019;mec.15088. 10.1111/mec.15088 31021499

[9] Maurice CF, Knowles SCL, Ladau J, Pollard KS, Fenton A, Pedersen AB (2015). Marked seasonal variation in the wild mouse gut microbiota. ISME J.

[10] Amato KR, Yeoman CJ, Kent A, Righini N, Carbonero F, Estrada A (2013). Habitat degradation impacts black howler monkey (Alouatta pigra) gastrointestinal microbiomes. ISME J.

[11] Belkaid Y, Hand TW (2014). Role of the microbiota in immunity and inflammation. Cell Elsevier.

[12] Williams CL, Caraballo-Rodríguez AM, Allaband C, Zarrinpar A, Knight R, Gauglitz JM (2018). Wildlife-microbiome interactions and disease: exploring opportunities for disease mitigation across ecological scales. Drug Discov Today Dis Models.

[13] Greenspan SE, Migliorini GH, Lyra ML, Pontes MR, Carvalho T, Ribeiro LP (2020). Warming drives ecological community changes linked to host-associated microbiome dysbiosis. Nat Clim Change.

[14] Apprill A, Robbins J, Eren AM, Pack AA, Reveillaud J, Mattila D, et al. Humpback whale populations share a core skin bacterial community: towards a health index for marine mammals? PLOS ONE. Public Library of Science; 2014;9:e90785. 10.1371/journal.pone.0090785 PMC3966734 24671052

[15] Loudon AH, Woodhams DC, Parfrey LW, Archer H, Knight R, McKenzie V (2014). Microbial community dynamics and effect of environmental microbial reservoirs on red-backed salamanders (Plethodon cinereus). ISME J.

[16] Bourne DG, Morrow KM, Webster NS (2016). Insights into the coral microbiome: underpinning the health and resilience of reef ecosystems. Annu Rev Microbiol.

[17] Hird SM, Ganz H, Eisen JA, Boyce WM. The cloacal microbiome of five wild duck species varies by species and influenza A virus infection status. Oh J, editor. mSphere. 2018;3:1–15. 10.1128/mSphere.00382-18 PMC6200988 30355662

[18] Stevens EJ, Bates KA, King KC. Host microbiota can facilitate pathogen infection. PLOS Pathog. Public Library of Science; 2021;17:e1009514. 10.1371/journal.ppat.1009514 PMC8118302 33984069

[19] Liévin-Le Moal V, Servin AL (2006). The front line of enteric host defense against unwelcome intrusion of harmful microorganisms: mucins, antimicrobial peptides, and microbiota. Clin Microbiol Rev.

[20] Huse SM, Welch DM, Morrison HG, Sogin ML (2010). Ironing out the wrinkles in the rare biosphere through improved OTU clustering: Ironing out the wrinkles in the rare biosphere. Environ Microbiol.

[21] Kembel SW, Wu M, Eisen JA, Green JL. Incorporating 16S gene copy number information improves estimates of microbial diversity and abundance. von Mering C, editor. PLoS Comput Biol. 2012;8:e1002743. 10.1371/journal.pcbi.1002743 PMC3486904 23133348

[22] Poretsky R, Rodriguez-R LM, Luo C, Tsementzi D, Konstantinidis KT. Strengths and limitations of 16S rRNA gene amplicon sequencing in revealing temporal microbial community dynamics. Rodriguez-Valera F, editor. PLoS ONE. 2014;9:e93827. 10.1371/journal.pone.0093827 PMC3979728 24714158

[23] Rosselló-Mora R, Amann R (2001). The species concept for prokaryotes. FEMS Microbiol Rev.

[24] Ellis JE, Missan DS, Shabilla M, Martinez D, Fry SE (2017). Rapid infectious disease identification by next-generation DNA sequencing. J Microbiol Methods.

[25] Walters W, Hyde ER, Berg-Lyons D, Ackermann G, Humphrey G, Parada A, et al. Improved bacterial 16S rRNA gene (V4 and V4–5) and fungal internal transcribed spacer marker gene primers for microbial community surveys. mSystems. 2016;1:e00009–15. 10.1128/mSystems.00009-15 PMC5069754 27822518

[26] Ellis JE, Missan DS, Shabilla M, Martinez D, Fry SE (2018). Microbial community profiling of peripheral blood in myalgic encephalomyelitis/chronic fatigue syndrome. Hum Microbiome J.

[27] R Core Development Team. R: A Language and Environment for Statistical Computing. Vienna, Austria: R Foundation for Statistical Computing; 2021.

[28] McMurdie PJ, Holmes S. Phyloseq: an R package for reproducible interactive analysis and graphics of microbiome census data. Watson M, editor. PLoS ONE. 2013;8:e61217. 10.1371/journal.pone.0061217 PMC3632530 23630581

[29] Oksanen J, Blanchet FG, Friendly M, Kindt R, Legendre P, McGlinn D, et al. Vegan: community ecology package. R package version 2.5–7. Httpcran R-Proj Org. 2020;

[30] Lahti L, Shetty S. microbiome R package. 2017; Available from: http://microbiome.github.io

[31] Duncan WW, Gehlbach FR, Middendorf GA. Nocturnal activity by diurnal lizards (Sceloporus jarrovi, S. virgatus) eaten by small owls (Glaucidium gnoma, Otus trichopsis). Carpenter GC, editor. Southwest Nat. 2003;48:218–22.

[32] Gehlbach FR, Gehlbach NY, Cartron J-LE (2010). Whiskered Screech-Owl (Megascops trichopsis). Raptors N M.

[33] Gehlbach FR, Stoleson SH. Western Screech-Owl (Megascops kennicottii). In: Cartron J-LE, editor. Raptors N M. Albuquerque, NM: University of Arizona Press; 2010. p. 511–23.

[34] Cannings R, Angell T, Pyle P, Patten M. Western screech-owl (Megascops kennicottii), version 1.0. In: Rodewald P, editor. Birds World. Ithaca, NY, USA: Cornell Lab of Ornithology; 2020.

[35] Mediannikov O, Sekeyová Z, Birg M-L, Raoult D. A Novel Obligate Intracellular Gamma-Proteobacterium Associated with Ixodid Ticks, Diplorickettsia massiliensis, Gen. Nov., Sp. Nov. Li W, editor. PLoS ONE. 2010;5:e11478. 10.1371/journal.pone.0011478 PMC2903611 20644718

[36] Subramanian G, Mediannikov O, Angelakis E, Socolovschi C, Kaplanski G, Martzolff L (2012). Diplorickettsia massiliensis as a human pathogen. Eur J Clin Microbiol Infect Dis.

[37] Merenstein C, Ward J, Allen D. *Diplorickettsia* Bacteria in an *Ixodes scapularis* Tick, Vermont, USA. Emerg Infect Dis. 2020 [cited 2022 Feb 2];26. Available from: http://wwwnc.cdc.gov/eid/article/26/5/19-1135_article.htm 10.3201/eid2605.191135 PMC7181933 32310073

[38] Corl A, Charter M, Rozman G, Toledo S, Turjeman S, Kamath PL (2020). Movement ecology and sex are linked to barn owl microbial community composition. Mol Ecol.

[39] Liu C, Finegold SM, Song Y, Lawson PA. Reclassification of Clostridium coccoides, Ruminococcus hansenii, Ruminococcus hydrogenotrophicus, Ruminococcus luti, Ruminococcus productus and Ruminococcus schinkii as Blautia coccoides gen. nov., comb. nov., Blautia hansenii comb. nov., Blautia hydrogenotrophica comb. nov., Blautia luti comb. nov., Blautia producta comb. nov., Blautia schinkii comb. nov. and description of Blautia wexlerae sp. nov., isolated from human faeces. Int J Syst Evol Microbiol. 2008;58:1896–902. 10.1099/ijs.0.65208-0 18676476

[40] De Cesare A, Sala C, Castellani G, Astolfi A, Indio V, Giardini A, et al. Effect of Lactobacillus acidophilus D2/CSL (CECT 4529) supplementation in drinking water on chicken crop and caeca microbiome. Yildirim A, editor. PLOS ONE. 2020;15:e0228338. 10.1371/journal.pone.0228338 PMC6980619 31978143

[41] Lysko SB, Baturina OA, Naumova NB, Lescheva NA, Pleshakova VI, Kabilov MR (2021). No-antibiotic-pectin-based treatment differently modified cloaca bacteriobiome of male and female broiler chickens. Agriculture.

[42] Liu X, Mao B, Gu J, Wu J, Cui S, Wang G (2021). *Blautia* —a new functional genus with potential probiotic properties?. Gut Microbes.

[43] Ellis JE, Missan DS, Shabilla M, Moschonas C, Saperstein D, Martinez D (2019). Comparison of the prokaryotic and eukaryotic microbial communities in peripheral blood from amyotrophic lateral sclerosis, multiple sclerosis, and control populations. Hum Microbiome J.

[44] Videvall E, Strandh M, Engelbrecht A, Cloete S, Cornwallis CK (2018). Measuring the gut microbiome in birds: Comparison of faecal and cloacal sampling. Mol Ecol Resour.

